# Low levels of vitamin C in dialysis patients is associated with decreased prealbumin and increased C-reactive protein

**DOI:** 10.1186/1471-2369-12-18

**Published:** 2011-05-06

**Authors:** Kunying Zhang, Li Liu, Xuyang Cheng, Jie Dong, Qiuming Geng, Li Zuo

**Affiliations:** 1 Renal Division, Department of Medicine, Peking University First Hospital, Peking, PR China; 2 Peking University Institute of Nephrology, Peking, PR China; 3 Key Laboratory of Renal Disease, Beijing, PR China; 4 Central Lab, Peking University Third Hospital; Beijing, 100034 PR China

## Abstract

**Background:**

Subclinical inflammation is a common phenomenon in patients on either continuous ambulatory peritoneal dialysis (CAPD) or maintenance hemodialysis (MHD). We hypothesized that vitamin C had anti-inflammation effect because of its electron offering ability. The current study was designed to test the relationship of plasma vitamin C level and some inflammatory markers.

**Methods:**

In this cross-sectional study, 284 dialysis patients were recruited, including 117 MHD and 167 CAPD patients. The demographics were recorded. Plasma vitamin C was measured by high-performance liquid chromatography. And we also measured body mass index (BMI, calculated as weight/height^2^), Kt/V, serum albumin, serum prealbumin, high-sensitivity C-reactive protein (hsCRP), ferritin, hemoglobin. The relationships between vitamin C and albumin, pre-albumin and hsCRP levels were tested by Spearman correlation analysis and multiple regression analysis.

Patients were classified into three subgroups by vitamin C level according to previous recommendation [1,2] in MHD and CAPD patients respectively: group A: < 2 ug/ml (< 11.4 umol/l, deficiency), group B: 2-4 ug/ml (11.4-22.8 umol/l, insufficiency) and group C: > 4 ug/ml (> 22.8 umol/l, normal and above).

**Results:**

Patients showed a widely distribution of plasma vitamin C levels in the total 284 dialysis patients. Vitamin C deficiency (< 2 ug/ml) was present in 95(33.45%) and insufficiency (2-4 ug/ml) in 88(30.99%). 73(25.70%) patients had plasma vitamin C levels within normal range (4-14 ug/ml) and 28(9.86%) at higher than normal levels (> 14 ug/ml). The similar proportion of different vitamin C levels was found in both MHD and CAPD groups.

Plasma vitamin C level was inversely associated with hsCRP concentration (Spearman r = -0.201, P = 0.001) and positively associated with prealbumin (Spearman r = 0.268, P < 0.001), albumin levels (Spearman r = 0.161, P = 0.007). In multiple linear regression analysis, plasma vitamin C level was inversely associated with log_10_hsCRP (P = 0.048) and positively with prealbumin levels (P = 0.002) adjusted for gender, age, diabetes, modality of dialysis and some other confounding effects.

**Conclusions:**

The investigation indicates that vitamin C deficiency is common in both MHD patients and CAPD patients. Plasma vitamin C level is positively associated with serum prealbumin level and negatively associated with hsCRP level in both groups. Vitamin C deficiency may play an important role in the increased inflammatory status in dialysis patients. Further studies are needed to determine whether inflammatory status in dialysis patients can be improved by using vitamin C supplements.

## Background

Because of its low molecular weight and high water solubility, vitamin C could be easily cleared from plasma during dialysis [3,4]. As important antioxidants, vitamin C was prominently consumed because of oxidative stress and inflammation in patients on dialysis, which could also cause low vitamin C level [5]. It is well established that plasma vitamin C level was generally low in patients on dialysis [3,6-8] compared with general population. In patients on dialysis, low plasma vitamin C level was associated with increased risk of cardiovascular morbidity and mortality [9].

Because of the bio-incompatibility, caused by membrane-blood contact and dialysate- blood contact, there were usually excess reactive oxygen species (ROS; e.g. hydroxyl radical, hydrogen peroxide and superoxide) production [10,11]. Furthermore, since various pro-inflammatory cytokines were promoted due to metabolic acidosis [12], volume overload [13], and non-sterile dialysate [14], patients were usually on mirco-inflammation status [15,16]. On the other side, antioxidant levels, such as plasma vitamin C level and reduced glutathione level, were usually decreased [3,5,17,18]. It was documented that inflammation was associated with increased risk of cardiovascular morbidity and mortality in patients on dialysis [19,20].

Evidences showed that low prealbumin level was associated with worse survival in patients on dialysis [21,22]. It was also reported that inflammation caused low prealbumin level [23,24]. But the relationship between plasma vitamin C and each of inflammatory markers and prealbumin was lacking, hence, we designed this study to pursue the relationship between vitamin C and inflammation.

## Methods

### Study patients

The study was designed as a cross-sectional analysis including both MHD patients and CAPD patients in Peking University First Hospital. Patients aged between 18 and 80 years and could provide informed consent were included. Patients who had autoimmune disease, malignancy, hepatitis in active conditions, currently used steroids or immune-suppressants, had positive human immunodeficiency virus(HIV) serology, and had any kind of acute infection within one month were excluded. However, patients having stable cardiovascular disease, or using central venous catheter, using angiotensin converting enzyme inhibitor (ACEI), angiotensin receptor antagonist (ARB), or statins were not excluded.

MHD patients were dialyzed three times per week, 4-4.5 hours per session, among them, 54 patients were treated with conventional hemodialysis (HD), 51 with high-flux hemodialysis (HFD), and 12 with hemodiafiltration (HDF). CAPD patients received 3-4 daily exchanges of 2L of peritoneal dialysis solution with either 1.5% or 2.5% glucose.

MHD and CAPD patients were classified by vitamin C level into three subgroups, respectively, according to previous recommendation [1,2]: group A, or vitamin C deficiency group, had vitamin C < 2 ug/ml; group B, or vitamin C insufficiency group, had vitamin C level between 2-4 ug/ml and group C, or vitamin C normal or above the normal group, had vitamin C > 4 ug/ml.

This study was approved by the local ethics committee and written informed consent was obtained from each participant.

### Sample collection and laboratory procedures

Blood samples for vitamin C determination were drawn from the arteriovenous fistula just before dialysis session in MHD patients, and from a forearm vein in CAPD patients. All subjects were in fasting condition. Blood was collected into heparin-containing tube, and then was transported in an ice bath to the laboratory. Plasma was separated by centrifugation (2000 × g, 10 min) at 4°C within 30 minutes, an aliquot of 200 ul plasma was mixed with 200 ul of 10% metaphosphoric acid (MPA) and stored at -80°C until analysis within 2 weeks.

Vitamin C was determined by high-performance liquid chromatography (HPLC) (Agilent 1100 series, Agilent Technologies, USA) on a Diamonsil C18 column(150 mm × 4.6 mm, 3 um) at 25°C. For HPLC analysis, the thawed plasma with MPA was centrifuged at 22000 g for 10 min, 4°C, 20 ul supernatants was injected into the HPLC by an autosampler. The mobile phase consisted of 25 mM sodium dihydrogenphosphate pH 2.6, the flow rate was 0.5 mL/min, and observed wavelength was 244 nm. Intra-assay and inter-assay coefficients of variation were 2.7% and 2.5%, respectively. The reference range of vitamin C level in normal population for this assay was 4-14 mg/L [1,2].

Prealbumin, high-sensitivity C-reactive protein (hsCRP), albumin, ferritin, calcium, serum bicarbonate, uric acid and hemoglobin were measured by standard techniques (the Clinical Laboratory of Peking University First Hospital, Beijing, China).

### Statistical Analysis

The relationships between vitamin C and each of albumin, prealbumin and hsCRP levels were tested using Spearman correlation analysis. Levels of each of vitamin C, hsCRP, albumin, prealbumin, uric acid, and hemoglobin were compared between MHD and CAPD patients using t-test. The above parameters were also compared using one-way analysis of variance (1-way-ANOVA) among groups A, B and C in each of MHD and CAPD patients, respectively. Using each of prealbumin, albumin and log_10_hsCRP as outcome and vitamin C as predictor, three linear regression model were constructed to explore the relationship between categories of plasma vitamin C level(< 2 ug/ml,2-4 ug/ml and > 4 ug/ml) and each of prealbumin, albumin and log_10_hsCRP after gender, age, diabetes, modality of dialysis and other confounding effects were adjusted. Numeric parameters were assessed by χ^2 ^test. Median, range, percentiles and Kruskal Wallis Test were used for non-normally distributed variables. Statistical analysis was performed using SPSS version11.5 (SPSS, Inc., Chicago, IL, USA). P values < 0.05 were considered as statistically significant.

## Results

### Demographics

The demographics of all the included patients were listed in table 1. There were 117 MHD patients and 167 CAPD patients, with a mean age of 59.8 ± 13.8 years and a mean dialysis vintage of 41.8 ± 37.2 months (median 32, range 1-233 ).

**Table 1:** Baseline characteristics of the study population (n = 284)

Parameters	MHD patients (n = 117)	CAPD patients (n = 167)	P value
Age(year)	59.3 ± 13.9	60.2 ± 13.7	0.594
Gender			0.540
Male	45(38.46%)	71(42.52%)	
Female	72(61.54%)	96(57.48%)	
BMI	22.7 ± 4.3	23.5 ± 3.2	0.071
Diabetes	9/117(7.7%)	48/167(28.7%)	0.000
Prealbumin(mg/l)	285.8 ± 77.3	371.7 ± 94.3	0.000
Albumin(g/l)	38.9 ± 3.9	36.0 ± 4.2	0.000
Uric acid(umol/l)	381.2 ± 82.9	376. 6 ± 79.8	0.642
Haemoglobin(g/l)	110.0 ± 17.2	109.5 ± 12.3	0.806
bicarbonate (mmol/l)	22.0 ± 3.6	25.7 ± 3.2	0.000
SBP(mmHg)	145.1 ± 19.8	128.9 ± 17.7	0.000
	Median(IQR)	Median(IQR)	
Vitamin C(ug/ml)	2.9(1.6-5.5)	3.0(1.8-5.0)	0.549
hsCRP(mg/l)	2.6 (1.0-6.5)	2.8(1.0-8.2)	0.756
Ferritin(ng/ml)	377(246.9-634.1)	374(215-585)	0.530
Dialysis vintage(m)	49(20-88)	24(9-49)	0.000

**Abbreviation: **BMI: body mass index; SBP: systolic blood pressure; IQR: inter quartile range; hsCRP: high-sensitivity C-reactive protein

The primary causes of end stage of renal disease (ESRD) were chronic glomerulonephritis (n = 93), diabetic nephropathy (n = 57), interstitial nephropathy (n = 45), hypertensive nephrosclerosis (n = 33), polycystic disease (n = 10), lupus nephropathy (n = 2), chronic pyelonephritis (n = 1), and others (n = 43).

Compared with CAPD patients, MHD patients had lower prealbumin, lower serum bicarbonate, higher albumin, higher systolic blood pressure and longer dialysis vintage. No statistical significance was found in age, gender and body mass index between CAPD and MHD patients.

### Distribution of plasma vitamin C

Patients showed a widely distribution of plasma vitamin C levels. The median plasma level was 3.34 ug/ml and the range from the 25th to the 75th percentile [inter quartile range (IQR)] was 1.87-5.74 ug/ml. vitamin C deficiency was present in 95(33.45%) and insufficiency in 88 ( 30.99% ) of the dialysis patients. 73(25.70%) patients had plasma vitamin C levels within normal range and 28(9.86%) at higher than normal levels. There were 48 patients taking vitamin C supplements (median 100 mg/day, range 50-600 mg/day), among whom, 9 patients with daily vitamin C supplement 50 to 100 mg had plasma vitamin C level less than 4 ug/ml, and 26 patients with daily vitamin C supplement 300 mg or more had higher plasma vitamin C level than normal.

### Association of vitamin C and prealbumin, albumin, hsCRP

In the total dialysis patients, plasma vitamin C level was positively associated with prealbumin (Spearman r = 0.268, P < 0.001), albumin (Spearman r = 0.161, P = 0.007), and inversely associated with hsCRP (Spearman r = -0.201, P = 0.001), age (Spearman r = -0.233, P = 0.000). Prealbumin is negatively correlated with hsCRP (Spearman r = -0.331, P < 0.01). No significant association was found between vitamin C and each of gender, hemoglobin, KT/V, body mass index, ferritin, or dialysis vintage in our study.

In MHD group, compared with group A, groups B and C had significantly lower age, higher albumin, higher prealbumin, and lower hsCRP (table 2) while gender, body mass index, KT/V and dialysis vintage were comparable among the three subgroups (table 2). The similar trend was found in CAPD patients (table 2).

**Table 2:** Clinical characteristics of three subgroups according to different vitamin C levels in MHD and CAPD groups

		MHD group			CAPD group	
	
	group A	group B	group C	group A	group B	group C
Parameters	< 2 ug/ml(n = 44)	2-4 ug/ml(n = 32)	> 4 ug/ml(n = 41)	< 2 ug/ml(n = 51)	2-4 ug/ml(n = 56)	> 4 ug/ml(n = 60)
Age(year)	66.4 ± 11.3	55.6 ± 12.8^a^	54.5 ± 14.5 ^a^	64.9 ± 11.4	57.8 ± 13.7^a^	58.4 ± 14.6^b^
Gender(male/female)	20/24	13/19	12/29	26/25	23/33	22/38
BMI	22.6 ± 4.5	22.9 ± 4.4	22.5 ± 4.0	24.0 ± 3.3	23.0 ± 2.9	23.6 ± 3.3
KT/V	1.6 ± 0.3	1.6 ± 0.3	1.7 ± 0.2	1.8 ± 0.4	1.9 ± 0.3	1.9 ± 0.4
Diabetes	5/39	1/31	3/38	17/34	18/38	13/47
Vitamin C(ug/ml)	1.2 ± 0.5	2.9 ± 0.6 ^a^	17.9 ± 20.4 ^ac^	1.4 ± 0.4	3.0 ± 0.6 ^a^	10.0 ± 10.6^ac^
Prealbumin(mg/l)	250.5 ± 77.2	305.0 ± 72.3 ^a^	309.2 ± 68.0 ^a^	337.7 ± 83.7	371.6 ± 85.7 ^b^	400.2 ± 102.1^a^
hsCRP(mg/l)	3.7(1.5-11.6)	1.8(0.7-6.3) ^b^	2.0(0.4-5.1) ^b^	4.2(1.9-14.8)	2.1(0.9-6.3) ^b^	2.1(0.9-5.2)^b^
Albumin(g/l)	37.4 ± 4.1	40.6 ± 3.8 ^a^	39.3 ± 3.1 ^b^	34.7 ± 4.6	36.8 ± 3.5^a^	36.3 ± 4.2^b^
Uric acid(umol/l)	380.3 ± 77.2	397.6 ± 87.4	368.9 ± 82.8	358.1 ± 86.3	380.8 ± 68.6	388.4 ± 82.2 ^b^
Haemoglobin(g/l)	107.5 ± 18.7	117.5 ± 15.0 ^b^	106.7 ± 15.7 ^c^	109.4 ± 13.9	110.8 ± 11.8	108.4 ± 11.5
Ferritin(ng/ml)	412.7 ± 247.2	397.2 ± 209.4	524.5 ± 386.4	508.2 ± 330.7	372.0 ± 294.3 ^b^	427.6 ± 309.3
Dialysis vintage(m)	64.0 ± 48. 2	55.3 ± 37.8	53.3 ± 46.0	27.4 ± 23.1	31.1 ± 26.3	32.6 ± 26.5

^a^P < 0.01:compared with group A; ^b^P < 0.05:compared with group A; ^c^P < 0.01: compared with group B

Patients receiving HDF treatment had lower log_10_hsCRP value(0.04 ± 0.71) compared with patients receiving HD treatment(0.52 ± 0.64, P = 0.03). There was no statistical significance in each of plasma vitamin C level, albumin and prealbumin among three MHD modalities.

Compared with patients without oral vitamin C supplements, patients with vitamin C supplements had increased albumin(38.47 ± 3.28 g/L vs 37.00 ± 4.44 g/L, P = 0.04), not significantly high prealbumin (341.08 ± 65.39 mg/L vs 336.83 ± 101.74 mg/L, P = 0.79), and not significantly low log_10_hsCRP(0.28 ± 0.65 vs 0.41 ± 0.69, P = 0.21).

### Multivariate analysis for vitamin C effects on prealbumin and hsCRP levels

In the whole dialysis patients, after adjusting for gender, age, diabetes and modality of dialysis, systolic blood pressure, metabolic acidosis and use of statins, per category increase of vitamin C caused 0.098 ± 0.049 decrement of log_10 _(hsCRP), this corresponding to 1.25 (1.12, 1.40) mg/L decrement of hsCRP (P = 0.048), (table 3).

**Table 3:** Association between plasma Vitamin C level and each of prealbumin, hsCRP and albumin levels adjusted for cofounders in multiple linear regressions

	variable	Beta	95%CI		t value	P value
					
			lower	upper		
prealbumin	VitC(per category increase)	0.155	7.021	29.477	3.186	0.002
	age	-0.243	-66.011	-28.674	-4.970	0.000
	Diabetes	-0.162	-63.307	-15.387	-3.219	0.001
	gender	0.125	5.590	44.194	2.528	0.012
	modality	0.424	39.327	66.793	7.573	0.000
	hsCRP(mg/l)	-0.255	-2.888	-1.337	-5.340	0.000
	bicarbonate (mmol/l)	-0.038	-3.561	1.613	-0.738	0.461
	SBP(mmHg)	0.030	-0.335	0.627	0.594	0.553
	statins	0.175	17.612	59.441	3.611	0.000
Log_10_hsCRP	VitC(per category increase)	-0.117	-0.195	-0.001	-1.984	0.048
	age	0.295	0.249	0.569	5.023	0.000
	Diabetes	-0.009	-0.224	0.192	-0.152	0.879
	gender	0.089	-0.042	0.293	1.472	0.142
	modality	-0.071	-0.182	0.057	-1.030	0.304
	bicarbonate (mmol/l)	-0.081	-0.037	0.008	-1.295	0.196
	SBP(mmHg)	-0.220	-0.012	-0.003	-3.581	0.000
	statins	0.025	-0.142	0.220	0.421	0.674
albumin	VitC(per category increase)	0.065	-0.246	0.928	1.138	0.256
	age	-0.252	-3.138	-1.214	-4.432	0.000
	Diabetes	-0.214	-3.548	-1.035	-3.575	0.000
	gender	-0.013	-1.127	0.900	-0.219	0.827
	modality	-0.176	-1.699	-0.252	-2.641	0.009
	bicarbonate (mmol/l)	-0.085	-0.232	0.040	-1.381	0.168
	SBP(mmHg)	0.099	-0.004	0.046	1.659	0.098
	statins	0.097	-0.148	2.026	1.694	0.091

**Abbreviation: **Vit C: vitamin C; SBP: systolic blood pressure

VitC(by categories) deficiency 1, insufficiency 2, normal and above 3; Age(by categories) < 60y 0, ≥60y 1; Diabetes: non- Diabetes 0, Diabetes 1; gender: F 0, M 1; modality: HD 1, HDF 2, HFD 3, CAPD 4; statins: without statins 0, with statins 1

Plasma vitamin C was positively with prealbumin levels (P = 0.002) adjusted for hsCRP and above cofactors, prealbumin level was 58.49 ± 12.29 mg/L lower in patients with vitamin C deficiency compared with patients with normal vitamin C level.

However, there was no statistical significant association between plasma vitamin C and albumin (table 3).

## Discussion

In the present study, it was found that vitamin C deficiency and insufficiency was a great problem in our dialysis patients. The results showed that lower plasma vitamin C level was associated with lower prealbumin and higher hsCRP concentration both in MHD and in CAPD patients. These findings collectively suggest that vitamin C deficiency could be associated with the higher clinical inflammatory status in our patients.

Patients with active autoimmune disease, malignancy, hepatitis and acute infection were excluded, because these kinds of conditions could cause acute oxidative stress and acute change of hsCRP and vitamin C. Patients with stable cardiovascular disease, using central venous catheter, or on regular medication of ACEI, ARB or statins were not excluded, because we thought these conditions could not change the relationship between hsCRP and vitamin C.

We found in both MHD and CAPD groups plasma vitamin C level was inversely associated with age, patients with plasma vitamin C < 2 ug/ml(group A) had older age. And we also found higher hsCRP, lower albumin and lower prealbumin in such patients, in common with the results of some previous studies [25,26]. These results may due to a decreasing intake [27], and an increasing oxidative stress with ageing [28-30].

Numerous studies reported that low plasma level of vitamin C in dialysis patients is commonly found owing to loss during dialysis procedure, inadequate dietary intake, reduced tubular reabsorption and impaired metabolism [3,7,31-33], which was in consistent with our investigation.

Several clinical studies suggested a linkage between oxidative stress and inﬂammation in uremic individuals, but few had focused on the relationship between vitamin C status and inflammatory indicators, and, if analyzed, statistical relationship between vitamin C and each of hsCRP and prealbumin were not obtained in those previous investigations [4,34,35]. What we revealed in our study did not parallel these observations. One important reason is that most of the previous study patients had daily oral vitamin C supplementation during those investigations, which could change the inflammatory status. Second, differences in age, smoking status, dialysis vintage and proportion of diabetes in the study populations may be responsible for the different results.

We proposed that the association between vitamin C and inflammation markers observed in our study was explained by that inflammation and oxidative stress have certain internal relations. The oxidative products such as superoxide anion (O_2_^-^) and hydrogen peroxide (H_2_O_2_) during dialysis procedure could trigger inflammation in uremic patients [10]. On the contrary, oxidative stress may be further aggravated by inflammation through potentiating respiratory burst activation in monocytes and neutrophils [18]. Some investigations proved that hsCRP had a close correlation with thiobarbituric acid reactive substances(TBARS) [5], a marker of lipid peroxidation, and α-tocopherol, and inflammatory proteins comprised C-reactive protein, fibrinogen and α1-antitrypsin were significantly correlated with most markers of oxidative stress [36]. Jenny M et al [37] found that acute administration of vitamin C reduced oxidant stress levels and improved NO-mediated resistance vessel dilatation in renal failure. Kamgar M at al [38] also showed that MHD patients had a decrease trend in C-reactive protein compared with the baseline after given oral vitamin C supplement for 8 weeks although it did not get statistical significance. However, Fumeron C et al [39] in a small-scale prospective study included 33 MHD patients (19 patients with oral vitamin C 250 mg three times per week after dialysis sessions, 14 patients without oral vitamin C) did not reveal that vitamin C could ameliorate oxidative stress and inflammation within 2 months.

Prealbumin is a negative acute-phase protein synthesized primarily by the liver [40], its plasma level reflects the presence of an ongoing or resolving inflammatory condition just as well as C-reactive protein. Prealbumin serum concentrations may have parallel changes with protein and energy intakes [41] and inverse changes in inflammation [42], it was somewhat equally considered as both markers of nutritional status and inflammation. Mehdi Rambod et al [21] reported in a 798 MHD patients cohort study, prealbumin inversely correlated with hsCRP and Interleukin-6 (IL-6), the surrogates of inflammation, which is very much like our observations.

Lee EJ et al [43] reported that plasma vitamin C levels positively related to albumin with Serum ascorbic acid < or = 9 mg/L. In the present investigation, we also found plasma vitamin C positively related with albumin in Spearman correlation analysis, but the association did not exist after adjustment for sex, age, diabetes, mode of dialysis and other confounding effects. Several recent documents investigated that plasma vitamin C levels closely associated with hemoglobin [34], iPTH and alkaline phosphatase [1]. However, we did not get such findings in both univariate and multivariate analysis in our sample, which maybe partly explained by the different selective standard of study population.

Previous study demonstrated that the elimination of plasma vitamin C is similar in both conventional hemodialysis and on-line hemodiafiltration [44], which is in line with our findings. In our study, there was no difference in plasma vitamin C level among the three MHD modalities. These results may be due to its small molecule (176.1 Da), highly soluble characteristic in water and low protein-bound. Patients receiving HDF treatment had lower log_10_hsCRP value compared with patients receiving HD treatment in our investigation, this observation was in consistent with some other previous results [45,46].

## Conclusion

In conclusion, our cross-sectional observation indicated that vitamin C deficiency was prevalent in dialysis patients, and lower plasma vitamin C level was associated with lower serum prealbumin and higher hsCRP concentration which may be closely link to oxidative stress and inflammation in uremia. Further large-scale, long-term interventional clinical trials are required to validate if vitamin C supplementation can reduce inflammatory process and improve outcome of dialysis patients.

## Competing interests

The authors declare that they have no competing interests.

## Authors' contributions

KYZ participated in the design of the study, sampling procedure, and drafted the manuscript. LL participated in the design of the study and sampling procedure. XYC participated in the design of the study. JD participated in the design of the study. QMG participated in sampling procedure and performed some statistical analysis. LZ conceived the study, and participated in its design and coordination and performed statistical analysis. All authors read and approved the final manuscript.

## Pre-publication history

The pre-publication history for this paper can be accessed here:

http://www.biomedcentral.com/1471-2369/12/18/prepub
